# Effectiveness of two programs of intermittent ferrous supplementation for treating iron-deficiency anemia in infants: randomized clinical trial

**DOI:** 10.1590/S1516-31802008000600004

**Published:** 2008-11-06

**Authors:** Geraldo Gaspar Paes Leme Coutinho, Eny Maria Goloni-Bertollo, Érika Cristina Pavarino-Bertelli

**Keywords:** Anemia, Anemia, iron deficiency, Infant, Prevalence, Iron, Anemia, Anemia ferropriva, Lactente, Prevalência, Ferro

## Abstract

**CONTEXT AND OBJECTIVE:**

Low compliance among mothers regarding the treatment of anemic children using daily doses of ferrous sulfate administered at home has been reported. This study aimed to establish the effectiveness of weekly doses administered by mothers at home compared with weekly supplements administered directly by healthcare professionals, to reduce the prevalence of anemia.

**DESIGN AND SETTING:**

This was a randomized clinical trial at a public healthcare clinic in São José do Rio Preto, Brazil.

**METHODS:**

This iron supplementation study was carried out using two intervention groups. The sample population was 130 infants, randomly allocated to two groups of 65 children. All of them received 12 weekly doses of 25 mg of elemental iron, administered either in the public healthcare clinic or at their homes.

**RESULTS:**

Treatment compliance was shown in both groups. The prevalence of anemia among all of the children was 75% at the beginning of supplementation and 46.3% at the end of the period (P < 0.0005), corresponding to a reduction in the number of anemic children of 38.3%. The average increases in hemoglobin concentration levels were 0.75 g/dl and 0.65 g/dl, respectively for home interventions and healthcare clinic administration (P < 0.00005).

**CONCLUSION:**

Weekly supplementation of 25 mg of iron was proven to be efficient in reducing anemia, using interventions both at home and in healthcare clinics. Compliance among the mothers was achieved because weekly supplementation was easy to administer and had few side effects. The results showed that the treatment presented low cost and fast benefits.

**CLINICAL TRIAL REGISTRATION NUMBER:**

NCT00655408.

## RESUMO

**CONTEXTO E OBJETIVOS:**

Considerando que a literatura mostra baixa adesão de mães de crianças anêmicas ao tratamento medicamentoso com sulfato ferroso administrado no domicílio em doses diárias, o presente trabalho teve a finalidade de comparar a efetividade da suplementação semanal, realizada por profissionais de Unidade Básica de Saúde (UBS) ou no domicílio, pelas mães.

**TIPO DE ESTUDO E LOCAL:**

Estudo clínico aleatório, realizado em UBS de São José do Rio Preto, São Paulo.

**MÉTODOS:**

O estudo foi realizado com dois grupos de intervenção. A população deste estudo submetida à suplementação teve como amostra inicial 130 lactentes alocados aleatoriamente em dois grupos de 65 crianças. Para todas as crianças, a suplementação foi de 25 mg de ferro elementar, uma vez por semana, durante 12 semanas consecutivas, administrada em uma UBS do município ou no domicílio das crianças.

**RESULTADOS:**

O estudo mostrou adesão das mães ao tratamento das crianças em ambos os grupos estudados. A prevalência de anemia foi de 75% antes do início da suplementação e de 46,3%, após o término da suplementação para todas as crianças do estudo (P < 0,0005). A taxa de redução do número de crianças anêmicas foi de aproximadamente 38,3%. O aumento médio da concentração de hemoglobina foi de 0,75 g/dl e de 0.65 g/dl, respectivamente, para o grupo de intervenção domiciliar e o suplementado na UBS (P < 0,00005 para o aumento em ambos).

**CONCLUSÕES:**

A suplementação intermitente de 25 mg de ferro na administração domiciliar ou em UBS mostra-se igualmente eficaz na redução da prevalência de anemia. A adesão das mães provavelmente deve-se à facilidade de suplementação apenas uma vez por semana e à baixa ocorrência de efeitos colaterais. Os resultados mostram um tratamento com baixo custo e rápido benefício.

## INTRODUCTION

Nutritional anemia, according to the World Health Organization (WHO), is a state in which low blood hemoglobin concentration is a consequence of deficiency of one or more essential nutrients, for any reason. Anemia due to insufficient iron is known as iron-deficiency anemia. This deficiency is the most common nutritional disorder during childhood and it affects individuals not only in developing countries but also in industrialized nations.^1^

It has been estimated that one third of the population of 5.5 billion people worldwide are anemic: including 40% of children between 0-12 years old, 51% of pregnant women, 35% of all women and 30% of men.^1^ In Latin America, iron-deficiency anemia has been presented as a critical problem for public health systems, affecting 46% of children and 23% of women of childbearing age.^2^ In Brazil, research shows high rates of iron deficiency in the populations of several states.^3-10^

The main factors implicated in the etiology of anemia during early childhood are iron reserves at birth, speed of growth, diet and the rate of iron loss.^11^ Reduction of blood hemoglobin concentrations impairs oxygen transport to tissues, thereby reducing cognitive functions, altering thermoregulation and affecting immunity.^12^ Research has shown that children with iron deficiency have lower performance in psychomotor tests compared with non-anemic children.^12,13^ For infants aged between six and 24 months, iron supplementation is the main treatment for iron deficiency. In this age range, the prevalence of anemia is at least 20%.^14^

The most common procedure for treatment and prophylaxis of anemic children attended in both private and public healthcare clinics has been to counsel mothers regarding iron supplementation at home. However, studies have shown a low rate of compliance among mothers over the recommended period. This may be caused by lack of care or, more likely, the side effects from long-term daily ferrous sulfate supplementation, which include nausea, vomiting, diarrhea, staining of teeth and abdominal pain.^15-18^ Some studies have shown satisfactory results in relation to reducing the prevalence of iron deficiency using weekly doses of ferrous sulfate,^4,19-22^ thereby avoiding these side effects.

## OBJECTIVE

This study aimed to compare the effectiveness of low-dose weekly ferrous sulfate supplementation administered to children in a public healthcare clinic in São José do Rio Preto, Brazil, with its administration by their mothers at home, in order to reduce the prevalence of anemia.

## PATIENTS AND METHOD

A clinical randomized study involving two intervention groups was carried out between April 2003 and January 2004. The development of this study followed the ethical requirements and all regulatory policies involved in research on humans, as laid down by Resolution 196/96 of the National Health Council of the Ministry of Health, 1996. This study was approved by the Research Ethics Committee of Faculdade de Medicina de São José do Rio Preto (Famerp).

The research population was initially 173 children aged 6 to 24 months who were registered at a public healthcare clinic in the district of Solo Sagrado in São José do Rio Preto. From this initial sample, 43 children were excluded from the study in accordance with specific predefined criteria such as the presence of an infectious process at the time of the first consultation or two weeks prior to the intervention,^23^ positive results from the Guthrie test,^24^ previous use of ferrous sulfate supplements and blood hemoglobin concentration of less than 7 g/dl.^25^ Iron supplementation was therefore started for 130 infants.

The subjects were randomly allocated to two intervention groups of 65 infants each by using a table of randomly distributed numbers. Sample size calculations indicated that, with ≥ 33 children per group, it would be possible to detect hemoglobin changes with a power of 0.8 and P value of 0.05, considering anemia prevalence of around of 60%^4^ and a reduction of 40%.^21^ The two groups included both anemic (serum hemoglobin concentration less than 11 g/dl)^1^ and non-anemic children. One group received weekly ferrous sulfate supplementation in the healthcare clinic and the other group received the same supplementation administered by their mothers or by another adult responsible for the child, at home.

Before starting the iron supplementation, during a private consultation, the parent or guardian of each child received a single initial explanation about the nature of the study, filled out a questionnaire to give information about the child and received instructions about the iron supplementation, according to the group to which each child was allocated. All parents or guardians of the children that participated in the study also signed written consent forms.

All of the infants underwent anthropometric evaluations and 2 ml of venous blood was drawn for blood tests (measurement of serum hemoglobin concentrations). In order to classify the children according to their anemic state, the criteria proposed by the WHO were adopted, i.e. identifying anemia when the hemoglobin values were less than 11.0 g/dl. Other studies^4,21,22^ have shown that the main cause of anemia in under-24-month-old children is iron deficiency, and therefore the hemoglobin concentration was used to define the prevalence of anemia, without evaluating organic mineral reserves (serum ferritin).

In order to evaluate the children's nutritional state, their weight (kg) and height (cm) measurements were taken before and after the period of iron supplementation. Weight and height data from the National Center for Health Statistics (NCHS) in the United States,^26^ were taken as the references.

The dose of ferrous sulfate was chosen based on two studies of weekly iron supplementation. In a study by Palupi et al.,^20^ weekly supplements of 30 mg of elemental iron resulted in a significant increase in hemoglobin among children aged two to five years old. In another study, Thu et al.^21^ used doses of 20 mg of elemental iron supplemented by vitamin C to improve iron absorption in children aged six to 24 months. In the present study, for both groups, a weekly dose of 1 ml of ferrous sulfate oral solution (Fer-in-Sol^®^) was used, corresponding to 25 mg of elemental iron. Thus, this was a dose lower than 30 mg, because the children were younger than those of Palupi at al.,20 but higher than 20 mg because the children did not receive supplementary vitamin C as in the study of Thu et al.^21^

In the thirteenth week of the trial, i.e. one week after the final dose, each child was again assessed using the same blood tests as used initially, to measure blood hemoglobin concentrations. Hence, the prevalence of anemia was calculated at the beginning and the end of the treatment period.

All the mothers of the children in both groups were asked to record the dates on which the weekly supplements were administered and also any occurrences of side effects, on a standard questionnaire that was similar to a vaccination card.

### Statistical analysis

In order to analyze the anthropomorphic data, the Epi-Info (version 6.04)^27^ statistical package was used. Descriptive statistics were used to analyze data relating to the effects of ferrous sulfate supplementation on the children in both groups. To compare means between three classes, analysis of variance (ANOVA) was used. To compare means between pairs of groups, the t-test for two samples was used, and to compare the likelihood of events in both groups, the test for normal approximation of two proportions was used or the McNemar test when recommended. The statistical analyses were performed using the Minitab software, version 12.22. The statistical significance level was defined as an alpha error of 5% (P-value < 0.05).

## RESULTS

The study showed compliance by the mothers in both study groups. Only 22 children (16.9%) did not complete the full 12 weeks of treatment, and this occurred because the mother or guardian had difficulties in traveling to the healthcare clinic or did not have time to take the child; or because the family moved to another region or there was a lack of interest in continuing the treatment. Hence, 108 children completed the entire treatment program, of whom 56 were treated at home and 52 in the public healthcare clinic.

The characteristics of the study population according to mean age, gender and anthropomorphic data (weight and height) are shown in Table 1. Statistical analysis comparing the two groups did not identify any significant differences in the variables analyzed (P > 0.05).

**Table 1:** Characteristics of the 108 children at the beginning of the study according to treatment location (home or healthcare clinic), mean age, gender and anthropometric data (weight and height)

Characteristics	Home supplementation (n = 56)	Healthcare clinic supplementation (n = 52)	P-value
Mean age (in months)	15.45 (SD = 5.80)	15.58 (SD = 5.64)	0.92
Weight at start of study (in kg)	10.01 (SD = 1.60)	9.96 (SD = 1.65)	0.87
Weight at birth (in kg)	3.22 (SD = 0.45)	3.14 (SD = 0.36)	0.27
Height (in cm)	76.81 (SD = 6.69)	77.70 (SD = 7.19)	0.51
Boys	55.36%	50.50%	0.58

*SD = standard deviation.*

The mean number of doses taken by the children in the study was 11 (standard deviation, SD = 2.79) out of a possible 12 for the complete period. Side effects such as vomiting and diarrhea were observed in only 4% of the children. With regard to these data, no significant differences were observed between the two groups (P = 0.32).

**Figure 1:**
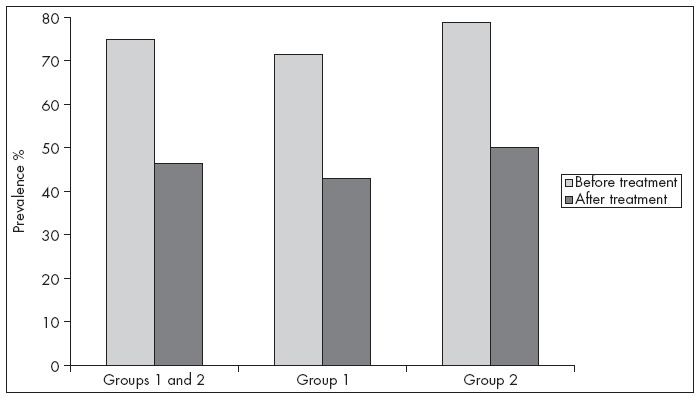
Prevalence of anemia among the 108 infants as percentages, before and after supplementation (respectively, in gray and black): Group 1 represents the children who received supplementation at home and Group 2, the children who received supplementation in the public healthcare clinic.

The data relating to the prevalence of anemia in the 108 infants before and after the treatment program are summarized in Figure 1. Before treatment, anemia was identified in 81 (75%) of the 108 children, of whom 41 were attended in the clinic and 40 at home. The anemia rate at the end of the treatment was 46.3%, corresponding to a reduction of around 38.3% (P < 0.0005). Considering each group separately, for the infants supplemented at home, the initial prevalence of 71.4% decreased to 42.8% at the end of the treatment. For the children supplemented in the clinic, the prevalence dropped from 78.8% to 50%. Significant reductions occurred in both groups (P < 0.0005). On the other hand, comparison of the reductions between the two groups did not identify any significant differences (P = 0.35).

At the end of the supplementation period, a significant increase of 0.70 g/dl (SD = 0.91) in mean hemoglobin concentrations was found (P < 0.00005). When considering the two groups separately, the initial and final mean blood hemoglobin concentrations were as presented in Table 2. Once again, no significant differences were found between the two groups (P = 0.13).

**Table 2:** Mean initial and final blood hemoglobin concentrations and change at the end of the supplementation program, for both groups of infants

	Initial mean hemoglobin (Hbi)		Final mean hemoglobin (Hbf)		Change		P-values
	g/dl	SD	g/dl	SD	Hbf - Hbi	SD	
Group 1 (n = 56)	10.32	1.29	11.07	1.02	+ 0.75	1.02	< 0.00005 P^1^
Group 2 (n = 52)	10.10	1.09	10.75	1.11	+ 0.65	0.79	< 0.00005 P^2^
P-value	P^3^ > 0.36		P^3^ > 0.13		P^3^ > 0.05		

*P^1^ = p-value for children supplemented at home; P^2^ = p-value for children supplemented in the public healthcare clinic; P^3^ = P-value between supplementation groups; Group 1: home supplementation children; Group 2: public healthcare clinic supplementation children; SD = standard deviation.*

Table 3 shows the increase in hemoglobin concentrations at the end of the supplementation period compared with the initial concentrations. The data shown in this table illustrate an inversely proportional relationship between blood hemoglobin concentration increases and the initial hemoglobin level. The children whose initial hemoglobin values were in the lower two categories (< 9.5 g/dl and between 9.5 and < 11.0 g/dl) presented significantly greater increases at the end of the intervention than did those in the third category (≥ 11.0 g/dl) (P < 0.02).

**Table 3:** Hemoglobin (Hb) change at the end of the intervention period, according to initial anemia

Initial degree of anemia	Change	
	Final mean Hb minus initial mean Hb	SD
Hb < 9.5 g/dl (n = 25)*	1.24	0.95
Hb between 9.5 and 11.0 g/dl (n = 59)*	0.83	0.73
Hb ≥ 11.0 g/dl (n = 24)	-0.03	0.82

*SD = standard deviation.*

* *The two first groups significantly differed from the third group regarding blood hemoglobin concentration change at the end of the intervention period (P < 0.02).*

## DISCUSSION

The results from this study showed that both programs were efficient in reducing anemia. There were no significant differences in the characteristics of intermittent iron supplementation using ferrous sulfate between administration in a public healthcare clinic and administration at home, with regard to reduction in anemia, increased blood hemoglobin concentrations, compliance with the treatment among the mothers and side effects.

Reduction of the occurrence of iron-deficiency anemia is a priority worldwide, especially among under-two-year-old children. This age range is the period of greatest brain development, as has been previously reported.^12^ In Brazil, because of the high prevalence of this disease, children's development is compromised. Over the last few decades, intermittent iron supplementation has emerged as a new therapy for iron-deficiency anemia.^4,19-22^

The present study showed treatment compliance in both groups: out of the 12 scheduled doses during the treatment period, an average of 11 doses was administered, and only 22 children (16.9%) out of the total of 130 did not complete the treatment. This result may have been achieved because the supplementation took place weekly, with the use of standard records to control administration of the doses. This procedure facilitated the treatment considerably because it significantly reduced the effort required from mothers. Furthermore, weekly supplementation reduced the incidence of side effects. In other studies that used daily supplementation with higher doses than in our study, compliance was low with drop-out rates ranging from 48% to 62%.^15-18^ In the present study the rate of reported side effects was low (4%).

Gillespie et al.^28^ recommended that the prophylactic doses of ferrous sulfate supplementation for children between six months and two years old should be 1 mg/kg/day. Since infants, on average, weigh approximately 7 kg at six months and 13 kg at two years of age, the total weekly doses would be 49 mg and 91 mg of iron, respectively.

In the present study, the significant reduction in the prevalence of anemia was achieved using single weekly doses of only 25 mg for 12 weeks, independently of the age or weight of the child, i.e. a dose that was lower than the prophylactic dose recommended. The same significant results were achieved in relation to the mean increase in blood hemoglobin concentrations. Other studies using intermittent supplementation have reported results that were similar to those of the present study,^20^ or results that were even better, through the use of higher iron doses, longer intervention periods and/or associated administration of vitamins and minerals.^21,22^

The mean increases in blood hemoglobin concentrations at the end of the intervention were more evident among infants who presented lower initial hemoglobin concentrations (lower than 9.5 g/l), i.e. among the most anemic children. On the other hand, when the initial concentrations indicated that there was no anemia (concentrations greater than or equal to 11.0 g/dl), there were even some decreases. The difference between the initial and final concentrations in the non-anemic group was significantly smaller than it was for children with some degree of anemia. This means that the organism efficiently absorbs iron according to its needs, and that the iron uptake may increase by up to 90% in severely anemic children.^29^

## CONCLUSIONS

In conclusion, simple and inexpensive programs of weekly iron supplementation can be used in public healthcare clinics or at home to effectively reduce the prevalence of iron-deficiency anemia. The results showed that both intervention programs described in this study fulfilled the requirements of providing easily administered iron supplementation and involving minimal human resources at low cost, because of the weekly doses.

## References

[1] World Health Organization (2001). Iron deficiency anaemia: assessment, prevention and control. A guide for programme managers.

[2] Ezzati M, Lopez AD, Rodgers A, Vander Hoorn S, Murray CJ, Comparative Risk Assessment Collaborating Group (2002). Selected major risk factors and global and regional burden of disease. Lancet.

[3] Monteiro CA, Szarfarc SC, Mondini L (2000). Tendência secular da anemia na infância na cidade de São Paulo (1984-1996). [Secular trends in childhood in the city of São Paulo, Brazil (1984-1996)]. Rev Saude Publica.

[4] Monteiro CA, Szarfarc SC, Brunken GS, Gross R, Conde WL (2001). Long-term preventive mass prescription of weekly doses of iron sulfate may be highly effective to reduce endemic child anemia. Food and Nutrition Bulletin.

[5] Nogueira-de-Almeida CA, Ricco RG, Del Ciampo LA, de Souza AM, Dutra-de-Oliveira JE (2001). Growth and hematological studies on Brazilian children of low socioeconomic level. Arch Latinoam Nutr.

[6] Osório MM, Lira PI, Batista M, Ashworth A (2001). Prevalence of anemia in children 6-59 months old in the state of Pernambuco, Brazil. Rev Panam Salud Publica.

[7] Hadler MC, Juliano Y, Sigulem DM (2002). Anemia do lactente: etiología e prevalência. [Anemia in infancy: etiology and prevalence]. J Pediatr (Rio J).

[8] Morais MB, Alves GM, Fagundes U (2005). Estado nutricional de crianças índias terenas: evolução do peso e estatura e prevalência atual de anemia. [Nutritional status of Terena indian children from Mato Grosso do Sul, Brazil: follow up of weight and height and current prevalence of anemia]. J Pediatr (Rio J).

[9] Silva LS, Giuglian ER, Aerts DR (2001). Prevalência e determinantes de anemia em crianças de Porto Alegre, RS, Brasil. [Prevalence and risk factors for anemia among children in Brazil]. Rev Saude Publica.

[10] Neves MB, da Silva EM, de Morais MB (2005). Prevalência e fatores associados à deficiência de ferro em lactentes atendidos em um centro de saúde-escola em Belém, Pará, Brazil. [Prevalence and factors associated with iron deficiency in infants treated at a primary care center in Belém, Para, Brazil]. Cad Saude Publica.

[11] Schmitz BAS, Picanço MR, Aquino KKNC (1998). Prevalência de desnutrição e anemia em pré-escolares de Brasília, Brasil. Pediatria Moderna.

[12] Walter T, De Andraca I, Chadud P, Perales CG (1989). Iron deficiency anemia: adverse effects on infant psychomotor development. Pediatrics.

[13] Hokama T, Gushi Ken M, Nosoko N (2005). Iron deficiency anaemia and child development. Asia Pac J Public Health.

[14] World Health Organization, United Nations Children's Fund, United Nations University (1996). Indicators for assessing iron deficiency and strategies for its prevention (draft based on a WHO/UNICEF/UNU Consultation, 6-10 December 1993).

[15] Palti H, Adler B, Hurvitz J, Tamir D, Freier S (1987). Use of iron supplements in infancy: a field trial. Bull World Health Organ.

[16] Romani SAM, Lira PIC, Batista M, Sequeira LAS, Freitas CLC (1991). Anemias em pré-escolares: diagnóstico, tratamento e avaliação; Recife, PE, Brasil. [Anemias in preschool children: diagnosis, treatment and evaluation; Recife, PE, Brazil]. Arch Latinoam Nutr.

[17] Torres MAA, Sato K, Juliano Y, Queiroz SS (1994). Terapêutica com doses profiláticas de sulfato ferroso como medida de intervenção no combate à carência de ferro em crianças atendidas em unidades básicas de saúde. [Treatment with prophylactic doses of ferrous sulphate in the fight against iron deficiency in children attended in basis health units]. Rev Saude Publica = J. Public Health.

[18] Szarfarc SC, Berg G, Santos ALS, Souza SB, Monteiro CA (1996). Prevenção de anemia no primeiro ano de vida em centros de saúde do município de Santo André, São Paulo. [Prevention of anemia in the first year of life in health centers of Santo André, São Paulo]. J Pediatr (Rio J).

[19] Liu X, Kang J, Zhao L, Viteri FE (1995). Intermittent iron supplementation is efficient and safe in controlling iron deficiency and anaemia in preschool children. Food Nutr Bulletin.

[20] Palupi L, Schultink W, Achadi E, Gross R (1997). Effective community intervention to improve hemoglobin status in preschoolers receiving once-weekly iron supplementation. Am J Clin Nutr.

[21] Thu BD, Schultink W, Dillon D, Gross R, Leswara ND, Khoi HH (1999). Effect of daily and weekly micronutrient supplementation on micronutrient deficiencies and growth in young Vietnamese children. Am J Clin Nutr.

[22] Ferreira MLM, Ferreira LOC, Silva AA, Batista M (2003). Efetividade da aplicação do sulfato ferroso em doses semanais no Programa Saúde da Família em Caruaru, Pernambuco, Brasil. [Effectiveness of weekly iron sulfate in the Family Health Program in Caruaru, Pernambuco State, Brazil]. Cad Saude Publica = Rep Public Health.

[23] Dallman PR (1996). Diagnóstico laboratorial da deficiência de ferro no lactente e na criança pequena. Anais Nestlé.

[24] Brasil. Ministério da Saúde Saúde da pessoa com deficiência. Portaria GM/MS no 822/GM de 06 de junho de 2001. Considerando a Portaria GM/MS nº 22, de 15 de janeiro de 1992, que trata do Programa de Diagnóstico Precoce do Hipotireoidismo Congênito e Fenilcetonúria.

[25] Camitta BM, Behrman RE, Kliegman RM, Arvin AM (1997). As anemias. Nelson: tratado de pediatria.

[26] World Health Organization (1995). Child growth standards. Physical status: the use and interpretation of anthropometry. Report of a WHO Expert Committee. Technical Report Series no 854.

[27] Dean AG, Dean JA, Burton AH, Dicker RC (1994). Epi-Info, version 6.02: a word processing, database and statistics program for public health.

[28] Gillespie S, Kevany J, Mason J (1991). Controlling iron deficiency.

[29] Pineda O, Ashmead HD (2001). Effectiveness of treatment of iron-deficiency anemia in infants and young children with ferrous bis-glycinate chelate. Nutrition.

